# Using a national level cross-sectional study to develop a Hospital Preparedness Index (HOSPI) for Covid-19 management: A case study from India

**DOI:** 10.1371/journal.pone.0269842

**Published:** 2022-07-27

**Authors:** Bhanu Duggal, Mona Duggal, Aparna Panch, Mithlesh Chourase, Praveen Gedam, Pushpendra Singh, Sujata Saunik, Lakshminarayan Subramanian

**Affiliations:** 1 Department of Cardiology, AIIMS Rishikesh, Rishikesh, Uttarakhand, India; 2 NITI AAYOG, New Delhi, India; 3 Post Graduate Institute of Medical Education and Research, Chandigarh, India; 4 Velai Analytics India Pvt Ltd, Hyderabad, India; 5 AIIMS Rishikesh, UK, India; 6 NHA, New Delhi, India; 7 IIIT Delhi, New Delhi, India; 8 Takemi Program in International Health, Harvard T.H. Chan School of Public Health, Boston, Massachusetts, United States of America; 9 Computer Science, Courant Institute of Mathematical Sciences, NYU and Population Health, NYU Grossman School of Medicine, New York City, New York, United States of America; 10 Velai Inc., Hyderabad, India; Norwegian Refugee Council, JORDAN

## Abstract

**Background:**

We developed a composite index–hospital preparedness index (HOSPI)–to gauge preparedness of hospitals in India to deal with COVID-19 pandemic.

**Methods:**

We developed and validated a comprehensive survey questionnaire containing 63 questions, out of which 16 critical items were identified and classified under 5 domains: staff preparedness, effects of COVID-19, protective gears, infrastructure, and future planning. Hospitals empaneled under Ayushman Bharat Yojana (ABY) were invited to the survey. The responses were analyzed using weighted negative log likelihood scores for the options. The preparedness of hospitals was ranked after averaging the scores state-wise and district-wise in select states. HOSPI scores for states were classified using K-means clustering.

**Findings:**

Out of 20,202 hospitals empaneled in ABY included in the study, a total of 954 hospitals responded to the questionnaire by July 2020. Domains 1, 2, and 4 contributed the most to the index. The overall preparedness was identified as the best in Goa, and 12 states/ UTs had scores above the national average score. Among the states which experienced high COVID-19 cases during the first pandemic wave, we identified a cluster of states with high HOSPI scores indicating better preparedness (Maharashtra, Tamil Nadu, Karnataka, Uttar Pradesh and Andhra Pradesh), and a cluster with low HOSPI scores indicating poor preparedness (Chhattisgarh, Delhi, Uttarakhand).

**Interpretation:**

Using this index, it is possible to identify areas for targeted improvement of hospital and staff preparedness to deal with the COVID-19 crisis.

## 1. Introduction

The COVID-19 pandemic highlighted the importance of prior surge planning as an invaluable component of hospital preparedness [1–3]. It is essential for hospitals to adequately equip their resources, including staff and infrastructure, for an effective response against such crises. Hospital staff training and screening for COVID-19 is crucial, because they come in close contact with COVID-19 patients [4, 5]. A planned and rapid upgradation of hospital infrastructure keeping in mind the specific needs of severe and critical forms of COVID-19 is necessary to control morbidity and mortality associated with the illness [6]. Usage of personal protective equipment (PPE) and proper training to the hospital staff in the same are indicators of better preparedness of a hospital [7]. Because of restricted access to healthcare delivery, the development and implementation of an effective telehealth and tele-triaging system can identify patients with priority and acute healthcare needs, thereby resulting in a better utilization of healthcare resources and limiting spread of the contagion [8].

Thus, the preparedness of a hospital against a pandemic is a multi-dimensional task which includes various domains and subdomains. This is especially important in a country like India, which has a diverse population spread across states with different geographies, population densities, education, standards of living, availability of healthcare facilities, and political diversity. With this background, we developed a novel index (the Hospital Preparedness Index, HOSPI), which helps to measure the extent of preparedness of a hospital and its staff in managing an emergency situation involving an infectious disease such as COVID-19. With the present cross-sectional pan-India study, we used the HOSPI to assess the level of preparedness of hospitals across various States and Union Territories (UTs) in India for managing the COVID-19 pandemic.

## 2. Materials and methods

### 2.1. Development and validation of the survey questionnaire

After obtaining permissions from the relevant authorities for the conduct of the study, a comprehensive survey questionnaire was developed to build an index to evaluate the extent of preparedness of hospitals and their staff in response to COVID-19 pandemic. The draft questionnaire was tested and validated by administering to a target sample of representatives from 10 hospitals, and the feedback received was used to further amend and update the questionnaire and options. The final approved version of the questionnaire had a total of 63 questions, which was a mix of open-ended questions (N = 16), questions with single best option (N = 39), and questions with multiple possible options (N = 8). For development of the hospital surge index, we excluded open ended questions, and included close ended questions with dichotomous (yes/ no) or scale like responses (16 questions).

The questions were categorized under four broad headings: general information about the hospital; impact of COVID-19 (impact on staff management, waste management, procurement activities, outpatient services, hospital practice activities, COVID-19 management and training, tele-triage and prioritization); and insights to improve services. The original questionnaire developed in English was also translated to Hindi to improve the convenience of answering. The dual-language questionnaire was adopted into a fillable online version using a custom software, and was rendered in both a web version and a smartphone version (android and iPhone platforms). The English language version of the actual questionnaire used in the study is available as **S1 Content**.

### 2.2. Conduct of the study

We collected the email IDs of all hospitals (both public and private) empaneled in the Ayushman Bharat Yojana (ABY or National Health Insurance Scheme) from the portal maintained by the National Health Authority (NHA) of India. As of April 2020, a total 20,347 hospitals were registered on the NHA portal under the ABY. All these empaneled hospitals were contacted by an email on May 10^th^ 2020; the email first described briefly the objectives and purpose of conducting the study, and a request to the hospitals for participating in the same, along with a link to the questionnaire. The person responsible for filling up the questionnaire could be one among the managing director/ chief executive officer/ head of the hospital, nodal officer appointed by the hospital for communication with the NHA. Considering the constraints and challenges of dealing with the ongoing pandemic, we fixed the ‘target sample size’ for responses as 5% of all hospitals from each state, which amounted to an overall minimum of 1,021 hospitals across the country. Considering the small number of hospitals in UTs, we kept the ‘target sample size’ for UTs as at least 1 large teaching hospital per UT.

The responsible persons from each hospital were requested to fill the questionnaire in the online mode using the link shared with them in the email within one week of receiving the email. To cater to the needs of places with poor internet connectivity, the smartphone version of the questionnaire was provided with an option for filling of responses offline and sending the filled questionnaire subsequently. After one week of sending the original email, a follow-up email was sent to the hospitals that did not respond to the first email. Apart from this, the survey team telephonically followed up with the non-responding hospitals to resolve any issues concerning the filling up of the survey questionnaire. In the case of rural areas where the hospitals often experience poor internet connectivity, the research team helped them in filling up the form through telephone interviews. The cut-off date for receiving filled-up questionnaires was fixed as 12^th^ July 2020.

### 2.3 Construction of the Hospital Preparedness Index (HOSPI)

The information collected from the study questionnaire was used to develop the HOSPI. At the stage of deliberation and discussion itself, 16 critical survey questions (variables) were identified out of the 63 questions in the validated questionnaire, and these 16 questions were categorized into five domains for representing the preparedness of the staff and hospital (**Table 1**).

**Table 1:** Domains and variables of the Hospital Preparedness Index, HOSPI.

No	Variables	Domain
1	Percentage of staff tested	Staff Preparedness
2	Staff screening	
3	Training of staff	
4	Donning off and donning on training (for PPE)	
5	Reduction in staff	Effects of COVID-19
6	Introduction of teleconsultation	
7	Reduction in revenue	
8	Staff adequacy for dialysis, cardiac, and ICU services	
9	Availability of PPE Kits	Protective Gears
10	PPE worn during OPD	
11	PPE worn in critical units	
12	Designated areas for COVID-19 screening	Infrastructure
13	Facilities for COVID-19 patients	
14	Equipped with medical devices	
15	Approach to resuming surgeries	Future Planning
16	Tele-triaging	

**Note:** ICU = intensive care units; PPE = personal protective equipment; OPD = outpatient department.

The identity of the 16 critical questions that were included in the HOSPI were not communicated to the responding hospitals to avoid bias in answering the questions. After receiving the filled questionnaire, the options for some of the 16 critical questions were modified and regrouped to better suit for analysis and quantification of the responses.

### 2.4. Data handling and statistics

For the analysis of the questionnaire responses and to provide hospital-wise, state-wise, and district- wise ranking of preparedness, we computed the weighted negative log likelihood score (WLS). For a given question, every possible response option for the question was weighted based on a negative log likelihood score based on the probability of selecting the response, and a static score set by an expert which ranged from 0.5 to 1.0 denoting a degree of COVID-19 preparedness for the chosen response. For all binary responses, we use two score values: a lower non-zero score of 0.5 was assigned to indicate the lower preparedness option and a score of 1 was assigned for the higher preparedness option. For questions with multiple options, the static score was marked on a scale of 0.5 to 1.0 for increasing levels of COVID-19 preparedness (S1 Table). Given the variability across the number of responses selecting each option, we weight each option using the negative log likelihood based on the probability of the response. Specifically, given a probability p of a given response to a specific question, we allocate a weight of log (1/(1-p)) for the response to indicate the negative log likelihood of not choosing a particular response, thereby preserving the relative popularity of a given response as well as a means to combine the negative log likelihood of the responses to all the questions in the survey. The negative log likelihood weight provides an entropy measure of a particular response as well as ensures the simple property that higher the probability of a response, higher the weight of the response. In addition, when combining the score across multiple survey questions, the negative log likelihood representation allows us to combine the weighted scores of the responses of multiple questions into a single score for pandemic preparedness by aggregating the weighted negative log likelihood scores of all the survey questions.

Assuming that a user chooses a response X for a question Q in the survey, the overall weight negative log-likelihood score (WLS) for the question is calculated as:

$$WLS(Q,X)=(Score(Q,X))\;x\;Log(1/(1‐P(Q,X)))/(\sum_{z}\;Score(Q,z))$$

where ‘Score (Q,X)’ is the score of the option X in question Q, ‘P(Q,X)’ represents the probability of users choosing the option X for the question Q, and ‘∑_*z*_ Score(Q,z)’ represents the sum of the scores of all the possible options for the question Q. The details of how the WLS was calculated for the question ‘Are the staff given training for donning on and donning off of PPE?’, which is the 4^th^ question in the HOSPI, is given in **Table 2**.

**Table 2:** Illustration of calculation of weighted negative log likelihood score for options used in the present study.

Question: Has any training been held for the surgeons and/or other staff in donning on and donning off PPE for performing surgeries/operations?				
Option	No of responses	Probability of selecting the option	Negative log likelihood score	Static Score assigned
Yes*	627	0.63141994	0.43346813	1
No	366	0.36858006	0.19968170	0.5

**Note:** PPE = personal protective equipment.

*favorable option indicating better preparedness.

This methodology was used to calculate the WLS for each option of the 16 included questions. Subsequently, the WLS allotted to the individual option selected by a hospital for the 16 questions were aggregated and a domain-wise score as well as an overall score was calculated for the hospital. The same exercise was repeated for all the hospitals included in the survey. Since the favorable response was given a higher score, a higher overall WLS indicates a higher level of preparedness in the concerned domain as well as an overall effect. For calculating state/ UT averages and district averages, the mean value of the total WLS was calculated, with the denominator being the total number of hospitals in the state/ UT/ district.

Overall score = ΣWLS score (% of staff tested + Staff screening + Training workers + Donning on and off training for PPE + Staff reduction + Introduction of tele-consultation + Reduction in revenue + Adequate of staff for Dialysis, Cardiac and ICU services + Availability of PPE kits + Types of PPE worn during OPD + Types of PPE worn during critical care surgery + Designated area for COVID-19 screening + Facilities for COVID-19 patients + Equipped with medical devices + Approach to resuming surgeries + Tele-triaging).

All data was collected electronically and was used for generating the WLS of each hospital, and to generate a state-wise and district-wise ranking of overall preparedness and domain-wise preparedness. Normalization of WLS and COVID-19 recovery rates was also performed using the same software. No comparative statistics were performed. The negative log likelihood weighting is a standard way used to combine scores across multiple diverse questions in a survey.

### 2.5. Clustering of the preparedness index components and COVID-19 cases at state level

K-Means Clustering is an unsupervised machine learning algorithm. In contrast to traditional supervised machine learning algorithms, K-Means attempts to classify data without having first been trained with labeled data. Once the algorithm has been run and the groups are defined, any new data can be easily assigned to the most relevant group [9].

For performing K-means cluster, “K” random points are selected as cluster centers (called centroids), and each data point is assigned to the closest cluster by calculating its distance with respect to each centroid. Next, the new cluster center is determined by computing the average of the assigned points. These steps are repeated until none of the cluster assignments change. To choose the correct number of “K” clusters points we graph the relationship between the number of clusters and Within ClusterSum of Squares (WCSS) then we select the number of clusters where the change in WCSS begins to level off (elbow method). WCSS is defined as the sum of the squared distance between each member of the cluster and its centroid [9].

Beyond this, there is no training or testing involved for any of the K-means clustering algorithms.

### 2.6. Ethical consideration and data availability

The study protocol was approved by the Institutional Review Board (IRB) of Indraprastha Institute of Information Technology, Delhi. Filling up of the questionnaire as such was voluntary for all the hospitals, and a basic consent was obtained from the hospitals as a part of the questionnaire itself. Identifiable patient information or human clinical data was not collected. All datasets leading to the results of the current study are available with the corresponding author on reasonable request.

## 3. Results

### 3.1. Study overview

The survey was conducted between May 2020 and July 2020. Considering the target sample of 1,021 hospitals, a complete response was obtained from 962/20,347 hospitals across India (4.7% overall response rate; 94.2% of the target sample size). A response rate of 90% and above of the target sample was achieved in 29 states and 3 UTs. No response was obtained from any hospital located in four UTs. Further, we excluded states which did not have a policy to implement ABY scheme in their state health machinery (namely, Odisha, Telangana, and West Bengal). Finally, for the ease of analysis, we combined the information obtained from 7 states from the North East Indian region which had returned responses from fewer than 15 hospitals. Thus, for the final analysis, we included information from a total of 954 hospitals from 26 states and 2 UTs **(S2 Table**).

Among the 954 responding hospitals the questionnaire was filled by the managing director or CEO of the hospital in 126 (13.2%) hospitals, by the surgeon/ physician/ public relations officer working as the nodal officer for NHA in 554 (58.1%) hospitals, and by administrative staff working for implementation of the ABY scheme in the remaining 274 (28.7%) hospitals. A total of 595 (62.3%) hospitals were public hospitals, 291 (30.5%) were private, for-profit hospitals, and 65 (6.8%) were private, not-for-profit hospitals; 3 hospitals did not respond to this question. A total of 63 (6.6%) hospitals were teaching hospitals, 823 (86.3%) were non-teaching hospitals, and 7 (0.7%) were central institutions; the remaining 61 hospitals did not respond appropriately to this question. The bed strength of the hospitals was < 50, 50–100, 100–200, and >250 in 609(63.8%), 206 (21.5%), 75(7.9%), and 48 (5.0%) hospitals respectively; 16 (1.7%) hospitals did not respond to this question.

### 3.2. Comparison of preparedness of hospitals among different States/ UTs in India

#### 3.2.1. Overall preparedness

**Fig 1:**
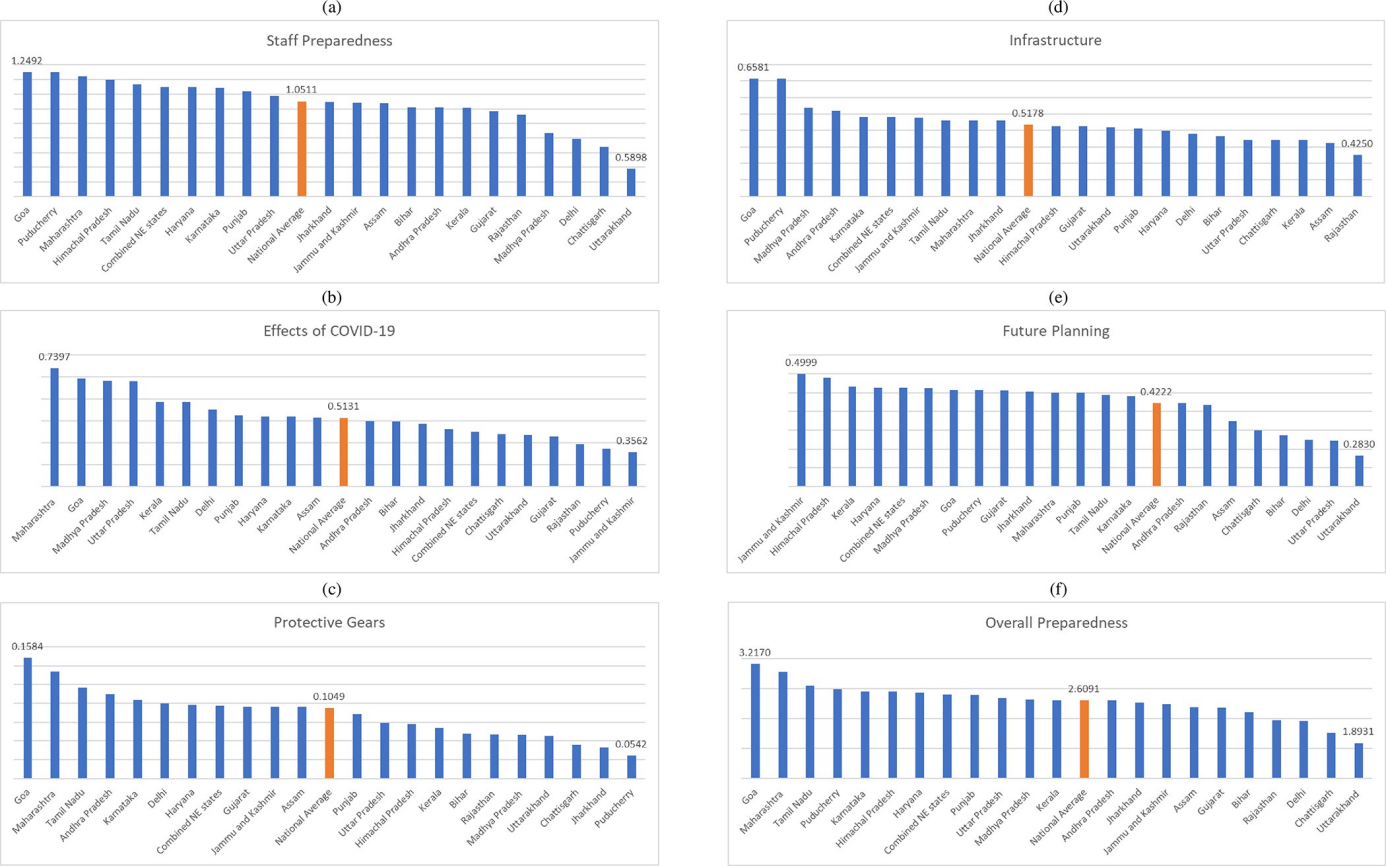
State-level comparisons of COVID-19 preparedness as per weighted negative log likelihood scores of the staff and hospital preparedness index. The national average is highlighted. (a): domain 1, staff preparedness; (b) domain 2, effects of COVID-19; (c) domain 3, protective gears; (d) domain 4, infrastructure; (e) domain 5, future planning, and; (f) overall preparedness.

The final overall preparedness index score achieved by the states/ UTs in India included in this study is summarized in **Table 3** and **Fig 1**. It was observed that domains 1, 2, and 4 (dealing with staff preparedness, effects of COVID-19, and infrastructure, respectively) contributed the most to the overall scoring (**S1 Fig**). The top three states/ UTs achieving the highest overall preparedness index scores were Goa, Maharashtra, and Tamil Nadu (**Fig 1F)**. The national average for the overall score was 2.6091, and this chronologically ranked between the 12^th^ and 13^th^ ranking entities, signifying that 12 states/ UTs in India were ‘more prepared’ and 10 states/ UTs were ‘less prepared’ than the national average, to combat the COVID-19 pandemic. The overall WLS of the best prepared state/UT (Goa, WLS = 3.2170) was 1.70 times higher than the total WLS of the least well-prepared state/UT (Uttarakhand, WLS = 1.8931).

**Table 3:** Domain-wise and overall weighted negative log likelihood scores and ranking of preparedness index by States/ UTs in India.

No	State/ UT	No of responding hospitals	Domain 1: Staff preparedness		Domain 2: Effects of COVID-19		Domain 3: Protective Gears		Domain 4: Infrastructure		Domain 5: Future Planning		Overall*	
			Score	Ranking	Score	Ranking	Score	Ranking	Score	Ranking	Score	Ranking	Score	Ranking
1	Goa	1	1.2492	#1	0.6942	#2	0.1584	#1	0.6581	#1	0.4571	#7	3.2170	#1
2	Maharashtra	41	1.2218	#3	0.7397	#1	0.1439	#2	0.5299	#9	0.4495	#11	3.0849	#2
3	Tamil Nadu	117	1.1684	#5	0.5860	#6	0.1264	#3	0.5304	#8	0.4435	#13	2.8547	#3
4	Puducherry	1	1.2492	#2	0.3721	#21	0.0542	#22	0.6581	#2	0.4571	#8	2.7907	#4
5	Karnataka	168	1.1447	#8	0.5197	#10	0.1137	#5	0.5406	#5	0.4400	#14	2.7588	#5
6	Himachal Pradesh	11	1.1974	#4	0.4631	#15	0.0877	#14	0.5130	#11	0.4890	#2	2.7502	#6
7	Haryana	27	1.1468	#7	0.5212	#9	0.1081	#7	0.4985	#15	0.4638	#4	2.7384	#7
8	Combined NE states^‡^	36	1.1485	#6	0.4488	#16	0.1078	#8	0.5402	#6	0.4629	#5	2.7082	#8
9	Punjab (including UT of Chandigarh)	39	1.1198	#9	0.5262	#8	0.0982	#12	0.5052	#14	0.4492	#12	2.6985	#9
10	Uttar Pradesh	53	1.0889	#10	0.6802	#4	0.0890	#13	0.4713	#18	0.3229	#21	2.6522	#10
11	Madhya Pradesh	30	0.8333	#19	0.6824	#3	0.0764	#18	0.5692	#3	0.4619	#6	2.6231	#11
12	Kerala	20	1.0064	#16	0.5869	#5	0.0838	#15	0.4707	#20	0.4669	#3	2.6147	#12
13	Andhra Pradesh	16	1.0088	#15	0.4984	#12	0.1197	#4	0.5605	#4	0.4214	#15	2.6089	#13
14	Jharkhand	47	1.0455	#11	0.4863	#14	0.0626	#21	0.5293	#10	0.4526	#10	2.5764	#14
15	Jammu and Kashmir	12	1.0430	#12	0.3562	#22	0.1065	#10	0.5382	#7	0.4999	#1	2.5439	#15
16	Assam	21	1.0393	#13	0.5147	#11	0.1063	#11	0.4615	#21	0.3739	#17	2.4957	#16
17	Gujarat	133	0.9817	#17	0.4272	#19	0.1065	#9	0.5130	#12	0.4551	#9	2.4834	#17
18	Bihar	43	1.0121	#14	0.4957	#13	0.0777	#16	0.4837	#17	0.3361	#19	2.4053	#18
19	Rajasthan	105	0.9585	#18	0.3942	#20	0.0772	#17	0.4250	#22	0.4169	#16	2.2718	#19
20	Delhi	5	0.7947	#20	0.5515	#7	0.1098	#6	0.4892	#16	0.3235	#20	2.2688	#20
21	Chhattisgarh	19	0.7402	#21	0.4392	#17	0.0659	#20	0.4711	#19	0.3495	#18	2.0658	#21
22	Uttarakhand	9	0.5898	#22	0.4350	#18	0.0753	#19	0.5100	#13	0.2830	#22	1.8931	#22
**23**	**National Average**	**954** #	**1.0511**	**NA**	**0.5131**	**NA**	**0.1049**	**NA**	**0.5178**	**NA**	**0.4222**	**NA**	**2.6091**	**NA**

UT: Union territory.

*Arranged in the ascending order of overall ranking of the states/ UTs

‡the combined states include Arunachal Pradesh, Manipur, Meghalaya, Mizoram, Nagaland, Sikkim, and Tripura

^#^total number of hospitals.

#### 3.2.2. Domain 1: Staff preparedness

A higher score in this domain represented better staff preparedness as a comprehensive indicator of the four sub-domains. Considering the national average score of 1.0511 for this domain, it was observed that 10 states/ UTs had scores above the national average, indicating that the remaining 12 states/ UTs had below-than-average staff preparedness. The WLS of the bestperforming state/UT in this domain (Goa, WLS = 1.2492) was 2.12 times higher than the WLS of the worst performing state/UTin this domain (Uttarakhand, WLS = 0.5898), indicating significant difference between the two ends of the spectrum with respect to the staff preparedness (**Table 3, Fig 1A**).

#### 3.2.3. Domain 2: Effects of COVID-19

A higher score in this domain indicated that the hospital and its staff were impacted to a lesser extent due to COVID-19, which in turn indicated better COVID-19 preparedness. Considering the national average score of 0.5131 for this domain, it was observed that 11 states/ UTs had scores above the national average, indicating that the remaining 11 states/ UTs had been affected worse due to COVID- 19 than the national average. The WLS of the best performing state/UT in this domain (Maharashtra, WLS = 0.7397) was 2.08 times higher than the WLS of the worst performing state/UT in this domain (Jammu and Kashmir, WLS = 0.3562) (**Table 3, Fig 1B**).

#### 3.2.4. Domain 3: Protective gears

A higher score in this domain indicated better availability and usage of protective gears by the hospital while managing COVID-19 suspected patients, which indicated better preparedness. Considering the national average score of 0.1049 for this domain, it was observed that 11 states/ UTs had scores above the national average, indicating that the remaining 11 state/ UTs were less prepared with respect to the availability and use of PPE kits during the pandemic than the national average. The WLS of the best performing state/UT in this domain (Goa, WLS = 0.1584) was 2.93 times higher than the WLS of the worst performing state/UT in this domain (Puducherry, WLS = 0.0542) (**Table 3, Fig 1C**).

#### 3.2.5. Domain 4: Infrastructure

A higher score in this domain suggested better preparedness of the hospital in terms of COVID-19 specific infrastructure. Considering the national average score of 0.5178 for this domain, it was observed that 10 states/ UTs had scores above the national average, indicating that the remaining 12 states/ UTs were less prepared with respect to developing COVID-19 specific infrastructure than the national average. The WLS of the best performing state in this domain (Goa, WLS = 0.6581) was 1.55 times higher than the WLS of the worst performing state in this domain (Rajasthan, WLS = 0.4250), indicating minimal difference between the different states in terms of availability of infrastructure for COVID-19 management (**Table 3, Fig 1D**).

#### 3.2.6. Domain 5: Future planning

A higher score in this domain indicated that the hospital and its staff had better future plans, thereby indicating better preparedness. Considering the national average score of 0.4222 for this domain, it was observed that 14 states/ UTs had scores above the national average, indicating that only 8 states/ UTs had future planning preparedness which was quantitatively below the national average. The WLS of the best performing state/ UT in this domain (Jammu and Kashmir, WLS = 0.4999) was 1.77 times higher than the WLS of the worst performing state in this domain (Uttarakhand, WLS = 0.2830), indicating that the future planning of almost all the considered states were not significantly different (**Table 3, Fig 1E**).

### 3.3. District level comparison of preparedness

We also used the WLS technique to evaluate the district-level preparedness of different states to combat COVID-19. For brevity, we are including the results of four states, namely Maharashtra, Tamil Nadu, Gujarat, and Rajasthan in the (**S2 Fig**).

### 3.4. Preparedness index cluster

**Fig 2:**
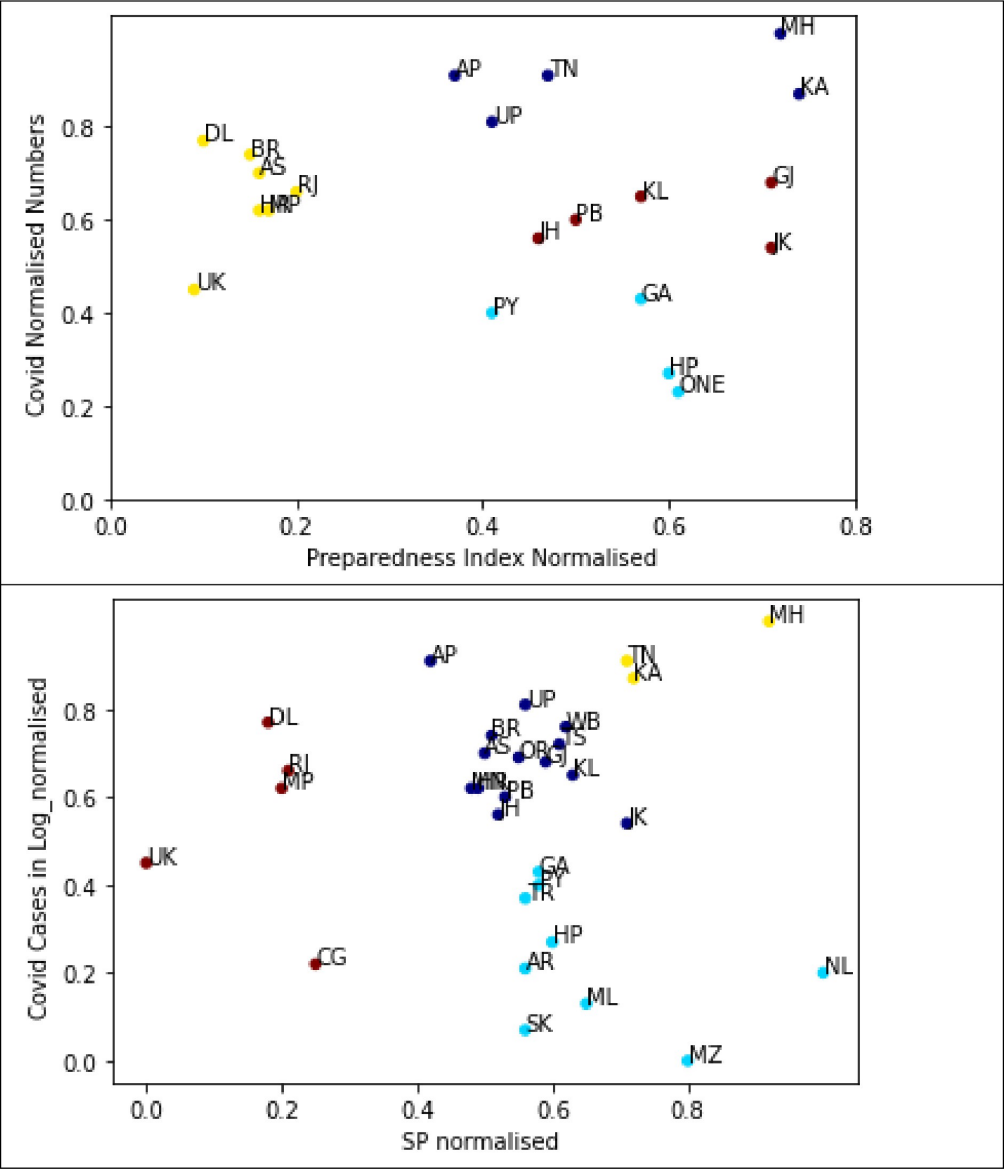
Clustering of states with different levels of preparedness when correlated with the number of active COVID cases in the state/ UT. (a) Overall HOSPI correlated with COVID cases; (b) Staff preparedness correlated with COVID cases.

**Fig 2A** represents the graphs that plot the HOSPI scores and COVID-19 cases of the states from which we had responses from. The states are grouped based on similar characteristics. The dark blue cluster represents the states Tamil Nadu, Karnataka, Andhra Pradesh, Uttar Pradesh, and Maharashtra, which were the worst affected by the pandemic (due to population density), but also have the highest preparedness index for the pandemic (better medical facilities). The dark red cluster contains the states having moderate impact of COVID-19 cases and moderate preparedness index (Jammu & Kashmir, Punjab, Kerala, Jharkhand, and Gujarat). The light blue cluster shows the majority of NE Indian states, along with Puducherry, Himachal Pradesh, and Goa, which also have very high preparedness index rate but lower cases compared to other states. Finally, the red cluster includes most of the North Indian states (Delhi, Rajasthan, Bihar, Uttarakhand) which had very low HOSPI score and experienced high COVID-19 cases.

**Fig 2B** represents graphs that plot the staff preparedness component of the HOSPI, of the states from which we had responses from. The states are grouped based on similar characteristics. The yellow cluster represents the states Tamil Nadu, Karnataka, and Maharashtra, which were the worst affected by the pandemic, but were also most prepared for the pandemic. The dark blue cluster contains the maximum number of states which had a moderate impact of COVID -19 cases, and moderate levels of staff preparedness. The light blue cluster shows the majority of NE Indian states which also had very high preparedness rate but comparatively lower cases. Finally, the red cluster is the North Indian states (Delhi, Chhattisgarh, and Uttarakhand) which were very under-prepared and also experienced high COVID-19 cases.

## 4. Discussion

This study represents our effort to quantitatively compare the extent of preparedness of hospitals in various states and UTs in India to combat the COVID-19 pandemic. The HOSPI is expected to enable policymakers to identify areas of concern and take appropriate steps to rectify them. Attempts have been made to assess hospital preparedness in the COVID-19 context in different areas in the world [10–13], and also to develop a vulnerability index for COVID-19 management and response in India [14]. To the best of our knowledge, ours is the first attempt to develop a comprehensive index for COVID-19 preparedness from the viewpoint of a hospital’s staff and infrastructure in India, and test the same in a pan-India setting.

The need for doing this study was first formulated in our minds three months into the pandemic situation, by which time the magnitude of the problem was increasingly becoming apparent. In the background of lack of a definitive, safe, and efficacious treatment for reduction of mortality or limiting infectivity of COVID-19 [15], and with an effective vaccine still being under development, stepping up of the preparedness of the hospitals, both public sector and private sector, was of extreme importance when we took up the study. The questionnaire that was developed as the first step in this exercise was therefore comprehensive and included as many as 63 questions across various domains. As for the sample size, considering the nature of the pandemic and the panic associated with it, we were expecting a low response rate from most of the states. Nevertheless, to convey the importance of the study, we had our team call up each hospital individually and explain the objectives of the survey. With such a proactive approach, we were able to achieve over 95% of our target sample size. Some states responded more proactively than others: the contribution of the caseload, level of motivation of the responsible person in each individual hospital, nature of the hospital services (i.e., public vs private, teaching vs non-teaching, etc), availability of staff to respond to the questionnaire, knowledgeability of the responding staff, and familiarity with internet-based surveys are some possible factors that have contributed to this disparity.

For the HOSPI, we chose 16 critical areas categorized into 5 important domains that we believe are the best indicators of the preparedness of a hospital. The first domain, staff preparedness, is critical because protection of the hospital staff while continuing the patient care along with limiting the spread of the disease is observed to play a key role during the pandemic [16]. The second domain explores the preparedness of the hospitals in the backdrop of the impact of the pandemic on the availability of hospital staff and reduction in hospital revenue. Being at a high risk of contracting the infection induces an incredible amount of stress among the frontline healthcare workers. As a result, there is an impact on the overall health as well as psychological health of these staff, leading to stress, burnouts, and other job-related hazards [17, 18]. A reduction in hospital workforce due to these effects, and also due to absenteeism [19], might lead to an inadequacy of staff in critical areas such as dialysis units, ICUs, and cardiac care units. With physical distancing being in place, hospitals are increasingly keen on taking up teleconsultation for managing non-emergency cases [20]. Cancellation of elective procedures and reduction in outpatient flow have contributed to reduction in hospital revenues [21]. The third domain deals with availability and usage of PPEs in two essential areas in the hospital (outpatient and critical care areas). Suboptimal availability and usage of PPEs has been reported in the context of COVID-19, and research to evaluate PPE preparedness and standardize PPE guidelines have been called for [22]. The fourth domain is about the availability of infrastructure specific to COVID-19, specifically with respect to screening, triaging, and managing COVID-19 patients, and adequate supply of medicines, devices, and isolation facilities in the hospital. The fifth domain deals with the plans of the hospital for the future, and comprises two sub-domains, namely tele- triaging, and plans to resume elective surgeries, which are crucial elements with regards to resumption of normal hospital activities post-COVID [23–26].

The four domains included in our HOSPI are also representative of the four domains identified to evaluate ‘surge capacity’ of a hospital during mass casualties, namely staff, supplies, space, and system [27]. This concept has been often used, most recently to develop a hospital medical surge preparedness index in the USA [28]. In our index, ‘staff’ is represented by domain 1, ‘supplies’ and ‘space’ are represented by sub-domains contained in domains 3 and 4, and ‘system’ is represented by sub-domains contained in domains 2 and 4. In addition to these 4, we also have included a ‘future planning’ domain in our index. Considering the fact that the COVID-19 pandemic is a surge situation, the domains included in the novel HOSPI appear to be comprehensive and truly representative of preparedness of hospitals to combat the pandemic.

In developing this index, we used weighted negative log likelihood scoring for quantifying responses. Negative log likelihood scoring allows us to analyze the results of an observational study in a way that makes the distribution of baseline covariates similar among all the responding subjects. In observational studies, the logistic regression model is most often used to estimate negative log likelihood scores [29]. In the present study, the negative log likelihood scores were developed based on the probability of a responding hospital selecting an option.

We also observed clustering of some states when we correlated the overall HOSPI with the number of COVID-19 cases; a similar clustering was also observed when we considered the staff preparedness component of the HOSPI alone. This clustering indicates the ground-level preparedness of various states/ UTs, in the backdrop of the actual number of COVID-19 cases that were reported in the state/ UT. This information is valuable for identifying the states/ UTs which require maximum support for upgrading their preparedness, and also to track the number of cases vis-à-vis the preparedness of the states to ensure that the COVID-19 cases are handled efficiently.

Based on the findings of the present study, the preparedness of hospitals and staff across the states can be understood in a better way by the policymakers, and steps can be taken to meet the unmet needs to improve the preparedness. In this background, we offer the following recommendations:

- Steps to protect hospital staff from COVID-19 effects (including screening hospital staff, providing adequate PPEs, training the hospital staff for PPE usage and COVID-19 related precautions) needs significant ramping up across the country
- To enhance reach and to reduce risks of physical transmission, online training sessions should be considered
- Develop adequate infrastructure facilities for isolation and treatment of COVID-19 patients
- Hospitals should be motivated to develop teleconsultation facilities with a view of assisting the patients as well as a revenue-generating enterprise
- Development of tele-triage between the health care centers and hospitals should be taken up as a priority under the ABY, with a view to reshape the treatment among patients of all socioeconomic groups

The findings of our study have to be interpreted in the light of certain limitations. Firstly, this was a questionnaire-based survey, which was filled in by the hospital representatives. The possibility of the hospitals reporting exaggerated claims to cover up certain deficiencies in the actual preparedness cannot be entirely ruled out. We also cannot confidently rule out the possibility of dishonest reporting as a ‘formality’ by less motivated hospital representatives. Because of lack of actual visit and physical assessment, the complete truthfulness of the responses cannot be reasonably assured, but hospitals wanted to reach out to the administration through this survey, when they really had deficiency of supplies, screening kits as they wanted NHA to give maximum coverage for covid patients. Next, there was a lack of uniformity in the number of hospitals that participated from each state/ UT considered. We wanted to do a time-bound survey representing all the states of India, and hence set a representative percentage of 5%, to understand the problems being faced by different states. The scores may not be representative of preparedness of all hospitals in a state, because of the restricted sample size selected to quickly gauge problems being faced by different geographical areas in India. We also included only those hospitals empaneled under the National health insurance scheme, to understand if special assistance was required by these hospitals under the national health insurance scheme. Inclusion of a higher number of hospitals might have improved the accuracy of the index in estimating the preparedness of the hospitals of a particular state. Future studies designed to evaluate the extent of usefulness of our novel index should be planned keeping in mind these limitations.

## 5. Conclusions

The index that we have developed is expected to help the hospitals and policymakers identify areas which are lagging behind in terms of preparedness and take necessary corrective actions for the same. This would also help in combating future waves of the COVID-19 pandemic, and also future pandemics, by proper utilization of resources including trained manpower and infrastructure.

## Supporting information

S1 Content Questionnaire used in the study. (PDF)

S1 Table State-wise distribution of sample size. (DOCX)

S2 Table Responses to the questions of the survey, with weights. (DOCX)

S1 Fig Relative contribution of each of the 5 domains towards the staff and hospital preparedness index. The maximum contribution is coming from domain 1 (staff preparedness, blue), domain 2 (effect of COVID-19, pink) and domain 4(infrastructure, orange).
(TIF)

S2 Fig District-wise comparison of preparedness among four states (Maharashtra, Gujarat, Rajasthan, and Tamil Nadu) across the 5 domains of the staff and hospital preparedness index. (a): domain 1, staff preparedness; (b) domain 2, effects of COVID-19; (c) domain 3, protective gears; (d) domain 4, infrastructure; and (e) domain 5, future planning.
(TIF)
