# Supplementary material for: Using a national level cross-sectional study to develop a Hospital Preparedness Index (HOSPI) for Covid-19 management: A case study from India
Source: PLoS One. 2022 Jul 27;17(7):e0269842. doi: 10.1371/journal.pone.0269842 (PMC9328545; doi:10.1371/journal.pone.0269842)
Supplement: S1 Content — (PDF) [file pone.0269842.s001.pdf]

Supplemental content 1: Questionnaire used in the study

Title of the Study: National Survey on Hospital Challenges and Needs during COVID-19 Pandemic

Objective and Consent: The objective of this survey is to understand every step of patient journey, hospital challenges, experiences, and feedback to provide better operational support and management to the empanelled hospitals under the Ayushman Bharat Yojana.

Your participation in this online (web/email) survey is voluntary. The platform is HIPPA compliant, high trust, and therefore, your data is safe and secure. All the information collected will be private, confidential and will not be revealed to any other third party. The survey involves online filling of information related to challenged and needs faced by hospitals during COVID-19 pandemic. The survey will take approximately 10-15 minutes. Please click on the agree button if you wish to participate in the survey.

[Agree/ Disagree]

Questionnaire

A. GENERAL INFORMATION

1. Name of the hospital
2. Name of the responder
3. Email of the responder
4. Qualification
5. Designation
6. Age
7. Your current location: state
8. Your current location: district
9. What is the nature of your practice?
   a. Private for profit
   b. Private not for profit
   c. Public not for profit
10. What is the bed capacity of your hospital?
   a. <50
   b. 50-100
   c. 100-250
   d. >250
11. Is your hospital a
   a. Teaching hospital
   b. Non-teaching hospital
   c. Central institute (autonomous, like AIIMS) hospital
   d. Na
12. Do you have digital patient health record system?
   a. Yes
   b. No
   c. In process

B (1) IMPACT OF COVID-19: GENERAL

13. What percentage change in the number of OPD patients have you observed during COVID-19 pandemic?
   a. <10%
   b. 10-19%
   c. 20-29%
   d. 30-39%
   e. 40-49%
   f. >50%
14. Is your hospital designated for treatment of especially COVID -19 patients?
   a. Yes
   b. No
15. Does your facility have designated screening and triaging area to review patients for COVID-19?
   a. Yes
   b. No

16. Do you have separate facilities for COVID-19 patients?
    a. Yes
    b. No
17. What areas have been does your facility have following designated for COVID-19?
    a. Isolation rooms
    b. Isolation ward
    c. Isolation wing
18. Do you have negative pressure rooms?
    a. Yes
    b. No

B (2) IMPACT OF COVID-19: STAFF MANAGEMENT, WASTE MANAGEMENT AND PROCUREMENT ACTIVITIES

19. Are you facing managerial or administrative challenges due to unavailability of administrative staff, nurses, or other staff members during COVID-19?
    a. Yes
    b. No
20. Do you see any reduction in number of the mentioned below staff? (multiple choice)
    a. Doctors
    b. Nurses
    c. Administrative staff
    d. Sanitation staff
21. If yes, what is the percentage reduction in number of above-mentioned staff during COVID-19?
    a. <10%
    b. 10-19%
    c. 20-29%
    d. 30-39%
    e. 40-49%
    f. >50%
22. Reduction in number of staff due to:
    a. Staff waylaid as reduction in revenues
    b. Quarantine
    c. Not willing to come
    d. Any other reason
23. Is there an adequate staff for essential services like dialysis, cardiac services, and ICU in your hospital?
    a. Yes
    b. No
24. Are you equipped in terms of supply of medical devices, medicines, and in-patient facilities for isolation of COVID-19 patients?
    a. Yes
    b. No
25. How many days of supply of personal protective equipment (PPE) does your hospital have?
26. Who is paying for personal protective equipment (PPE)?
    a. Hospital
    b. Procedure cost
    c. Others
    d. Not sure
27. Do you provide masks to patients and relatives when they visit hospital?
    a. Yes
    b. No
    c. On requirement
28. Does your hospital follow standard protocol for disposal of waste i.e., gloves, masks etc?
    a. Yes
    b. No
    c. Not Sure

B (3) IMPACT OF COVID-19: OUTPATIENT SERVICES, COVID-19 MANAGEMENT, TRAINING

29. Are you treating COVID-19 patients?
    a. Yes

    b. No

30. Have you been provided with any guidelines by the state regarding COVID-19 management?
    a. Yes
    b. No
31. Have you advised your staff to download *Aarogyasetu* app?
    a. Yes
    b. No
32. What extent has your healthcare workers been trained in diagnosing and treating COVID-19 patients?
    a. No training
    b. Minimal training (few staff or limited experience by trained staff)
    c. Moderate training (most of the staff but with limited experience by trained staff)
    d. Substantial training (most of the staff and at least moderate experience by trained staff)

[**NOTE**: We acknowledge that doctors, nurses, and support staff face high risk of infection during COVID-19 diagnosis and treatment in the hospital and therefore, may require periodic COVID-19 testing. Keeping this in mind, please answer the below questions]

33. Do you have a policy in-place of testing staff members for COVID-19?
    a. Yes
    b. No
    c. In process
34. If yes, what percentage of hospital staff members testing has been done at your hospital till May 15, 2020?
    a. <10%
    b. 10-19%
    c. 20-29%
    d. 30-39%
    e. 40-49%
    f. >50%
35. Who is paying for COVID-19 testing of the staff at hospital?
    a. Paid by staff
    b. Hospital management
    c. Other

B (4) IMPACT OF COVID-19: HOSPITAL PRACTICE ACTIVITIES

36. If the hospital is performing surgeries, has any training been held for the surgeons and/or other staff in donning on and donning off PPE for performing surgeries/operations?
    a. Yes
    b. No
37. Which of the following are you wearing in the OPD (multiple answer option)?
    a. Triple layer surgical mask
    b. Cloth mask
    c. N95 mask
    d. Goggles
    e. Face shield
    f. Shoe cover
    g. Cover All
38. Which of the following are you wearing in the critical care areas (multiple answer option)?
    a. Triple layer surgical mask
    b. Cloth mask
    c. N95 mask
    d. Goggles
    e. Face shield
    f. Shoe cover
    g. Cover All
39. If you are currently seeing non-COVID patients, what kind of patients are these (multiple answer option)?
    a. Emergency
    b. Obstetric Services
    c. OPD
    d. Elective surgeries
    e. Others

40. What surgeries are you currently performing (multiple answer option):
    a. Emergency
    b. Obstetric Services
    c. OPD
    d. Elective surgeries
    e. Others
41. Are you performing aerosols generating procedures?
    a. Yes
    b. No
42. If yes, what service are you taking?

B (5) IMPACT OF COVID-19: TELE- TRIAGE AND PRIORITISATION

43. Are you using tele-consultation for all patients?
    a. Yes
    b. No
44. If yes, what service are you taking
    a. Telephonic consultation
    b. Email/WhatsApp/ Other social media app
    c. Video consultation
    d. None as of now, but plan to start
    e. I do not plan to start any of it soon
45. From the information available, what is your opinion regarding risk of contracting COVID in cardiologists and assisting nurses?
    a. They are at the same risk level as other facility staff members
    b. They are at the reduced risk level than other facility staff members
    c. They are at the higher risk level than other facility staff members
46. Is there any percentage reduction in revenue due to COVID-19?
    a. Yes
    b. No
47. Upon easing of COVID-19, what would be your strategies or plan to regenerate the lost hospital revenue
    a. Restart elective surgeries as soon as lockdown lifts up
    b. Start elective surgeries after 1 week of lockdown upliftment
    c. Not sure when to start elective surgeries post lockdown
    d. Still in process of developing a strategy
48. Regarding surgeries that would be planned after resumption of clinical duty, what would be your preferred approach?
    a. Include testing for COVID-19 as a part of preoperative screening
    b. Use additional personal protective equipment (PPE) in all cases
    c. Performing testing for COVID-19 preoperatively AND use PPE in all cases
    d. Unsure of what to do, therefore awaiting clear guidelines

C. AYUSHMAN BHARAT YOJANA AND COVID-19

[Note: From last couple of years, many services in secondary and tertiary level hospitals are provided through Ayushman Bharat Yojana. Please answer the below questions to understand how these services could be redesigned in wake of COVID-19 situation.]

49. Do you know that testing and treatment of COVID-19 is covered under Ayushman Bharat?
    a. Yes
    b. No
50. Do you have any concerns that patients utilizing Ayushman Bharat Yojana, who typically belong to a lower socio-economic status, may have higher chances of having COVID-19?
    a. Yes
    b. No
51. Would you like Ayushman Bharat to support development of a tele-triage system between health & wellness centers and empanelled hospitals to prioritize, screen, treat and refer all patients who need intensive treatment to Ayushman Bharat empanelled hospitals?
    a. Yes
    b. No
52. How do you think Ayushman Bharat can support hospitals during COVID-19?
    a. Supply of PPE

b. Face masks, gloves for patients and staff
c. Ventilators
d. Postal home delivery of medicines
e. Tele-consultation and follow-up budget
f. Supply wearable devices for remote monitoring of patients' BP and pulse etc
g. Others

53. Have you discussed with local, state, tribal and regional planning contacts to determine the hospital's role in the COVID-19 pandemic?
    a. Yes
    b. No

D. INSIGHTS TO IMPROVE SERVICES

54. Have you modified your routine OPD protocol to treat patients with flu?
    a. Yes
    b. No
55. If yes, what are the measures taken (multiple choice question)?
    a. Thermal screening
    b. Swab testing
    c. Others
56. Do you think routine surgeries should be performed during COVID-19 pandemic?
    a. Yes
    b. No
57. If yes, please give your reasons:
58. If no, then how long do you think routine surgeries should be postponed?
59. If answer is no, which surgeries should be postponed?
60. As a healthcare provider, do you perceive any increased stress or anxiety or fear in your daily life during this COVID-19 pandemic?
    a. Yes
    b. No
61. If yes, what are your suggestions to help reduce the stress amongst healthcare providers?
62. How prepared do you feel to handle the COVID-19 situation?
63. If you feel any challenges are not discussed in the survey, please feel free to mention here.

Supplemental table 1: **State-wise distribution of sample size**

No	State/ UT	Total registered hospitals	Target (5% response)	Achieved	% achieved	Remarks
1	Andhra Pradesh	1779	89	16	18.0	Included
2	Arunachal Pradesh	20	1	1	100.0	Clubbed with NE states
3	Assam	390	20	21	105.0	Included
4	Bihar	871	44	43	97.7	Included
5	Chhattisgarh	334	17	19	111.8	Included
6	Goa	25	1	1	100.0	Included
7	Gujarat	2706	135	133	98.5	Included
8	Haryana	539	27	27	100.0	Included
9	Himachal Pradesh	215	11	11	100.0	Included
10	Jammu & Kashmir	230	12	12	100.0	Included
11	Jharkhand	757	38	47	123.7	Included
12	Karnataka	3423	171	168	98.2	Included
13	Kerala	405	20	20	100.0	Included
14	Madhya Pradesh	590	30	30	100.0	Included
15	Maharashtra	811	41	41	100.0	Included
16	Manipur	58	3	3	100.0	Clubbed with NE states
17	Meghalaya	179	9	9	100.0	Clubbed with NE states
18	Mizoram	101	5	5	100.0	Clubbed with NE states
19	Nagaland	101	5	5	100.0	Clubbed with NE states
20	Odisha	30	2	2	100.0	Excluded[1]
21	Punjab	771	39	39	100.0	Included
22	Rajasthan	2090	105	105	100.0	Included
23	Sikkim	12	1	1	100.0	Clubbed with NE states
24	Tamil Nadu	2334	117	117	100.0	Included
25	Telangana	21	1	1	100.0	Excluded[1]
26	Tripura	104	5	12	240.0	Clubbed with NE states
27	Uttarakhand	190	10	9	90.0	Included
28	Uttar Pradesh	1080	54	53	98.1	Included
29	West Bengal	61	3	4	133.3	Excluded[1]
30	Chandigarh	22	1	1	100.0	Clubbed with Punjab
31	Delhi	63	3	5	166.7	Included
32	Puducherry	24	1	1	100.0	Included
33	Lakshadweep	1	0	0	0.0	Excluded[2]
34	Andaman & Nicobar Islands	3	0	0	0.0	Excluded[2]
35	Dadar & Nagar Haveli	4	0	0	0.0	Excluded[2]
36	Daman & Diu	3	0	0	0.0	Excluded[2]
	**Total**	**20,347**	**1,021**	**962**	**94.2**	
	**Included in analysis**	**20,202**	**1,014**	**954**		
	**Excluded from analysis**	**145**	**7**	**8**		

Note: [1]Not participating in ABY; [2]No response

Supplemental figure 1: Relative contribution of each of the 5 domains towards the staff and hospital preparedness index. The maximum contribution is coming from domain 1 (staff preparedness, blue), domain 2 (effect of COVID-19, pink) and domain 4 (infrastructure, orange).

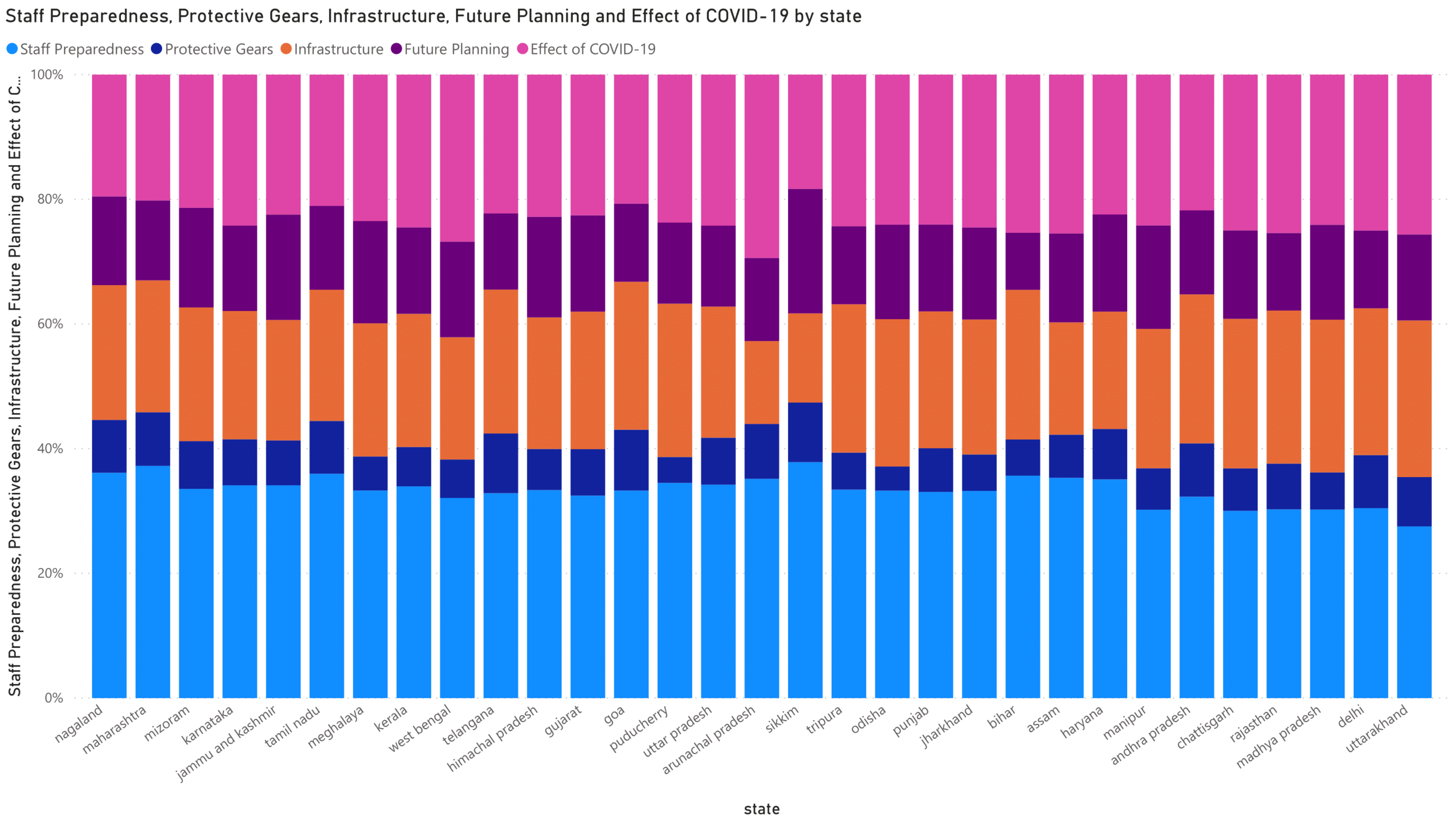

Supplemental figure 2: District-wise comparison of preparedness among four states (Maharashtra, Gujarat, Rajasthan, and Tamil Nadu) across the 5 domains of the staff and hospital preparedness index. (a): domain 1, staff preparedness; (b) domain 2, effects of COVID-19; (c) domain 3, protective gears; (d) domain 4, infrastructure; and (e) domain 5, future planning.

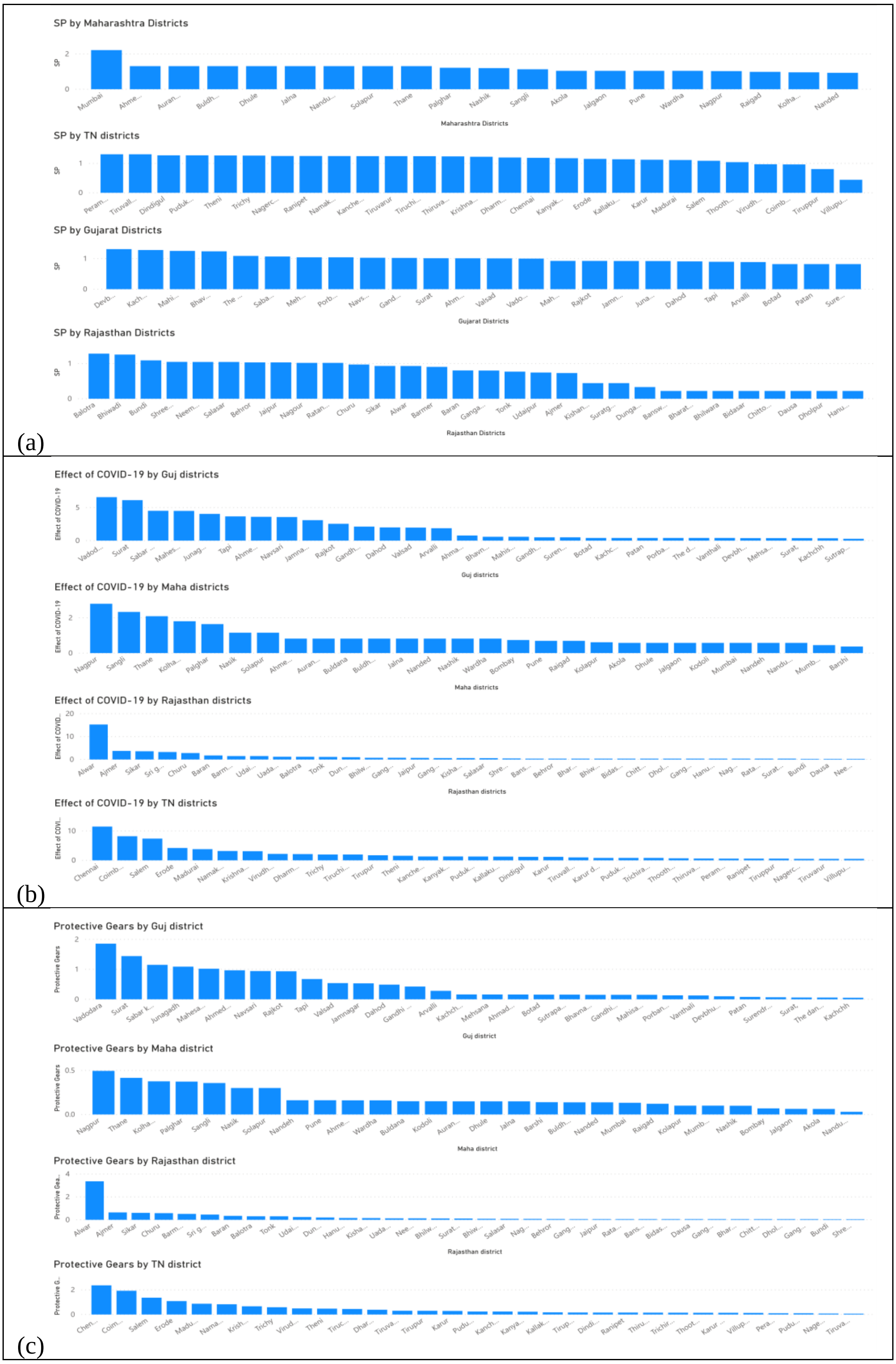

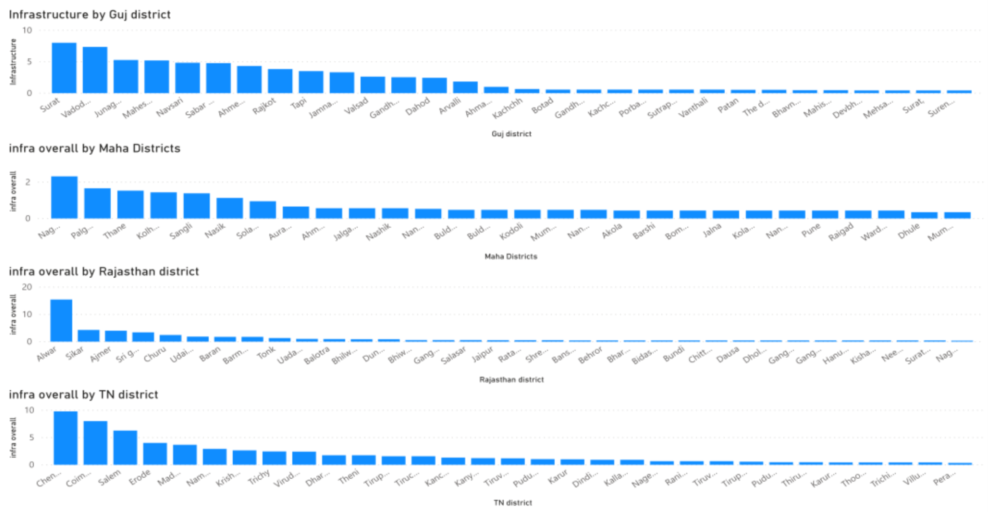
Infrastructure by Guj district
Guj district
infra overall by Maha Districts
Maha Districts
infra overall by Rajasthan district
Rajasthan district
infra overall by TN district
TN district

(d)

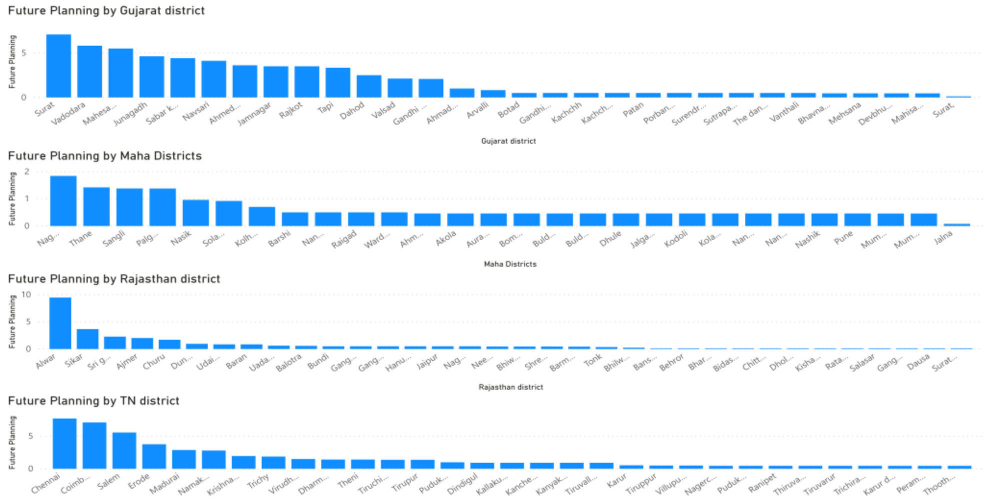
Future Planning by Gujarat district
Gujarat district
Future Planning by Maha Districts
Maha Districts
Future Planning by Rajasthan district
Rajasthan district
Future Planning by TN district

(e)
