# Supplementary figures and images for: Using a national level cross-sectional study to develop a Hospital Preparedness Index (HOSPI) for Covid-19 management: A case study from India
Source: PLoS One. 2022 Jul 27;17(7):e0269842. doi: 10.1371/journal.pone.0269842 (PMC9328545; doi:10.1371/journal.pone.0269842)

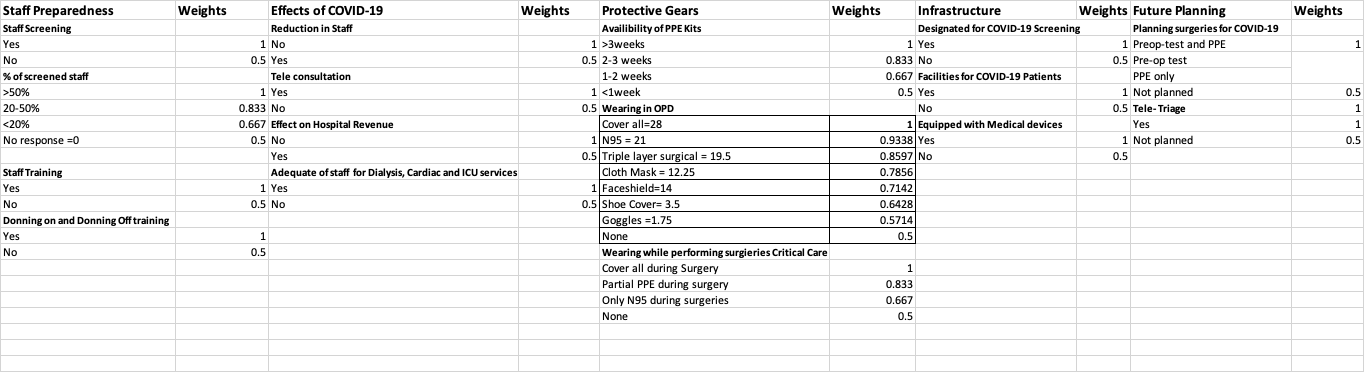


Supplementary table 1 (S1 )

Supplement: S1 Table — (DOCX) [file pone.0269842.s002.docx]

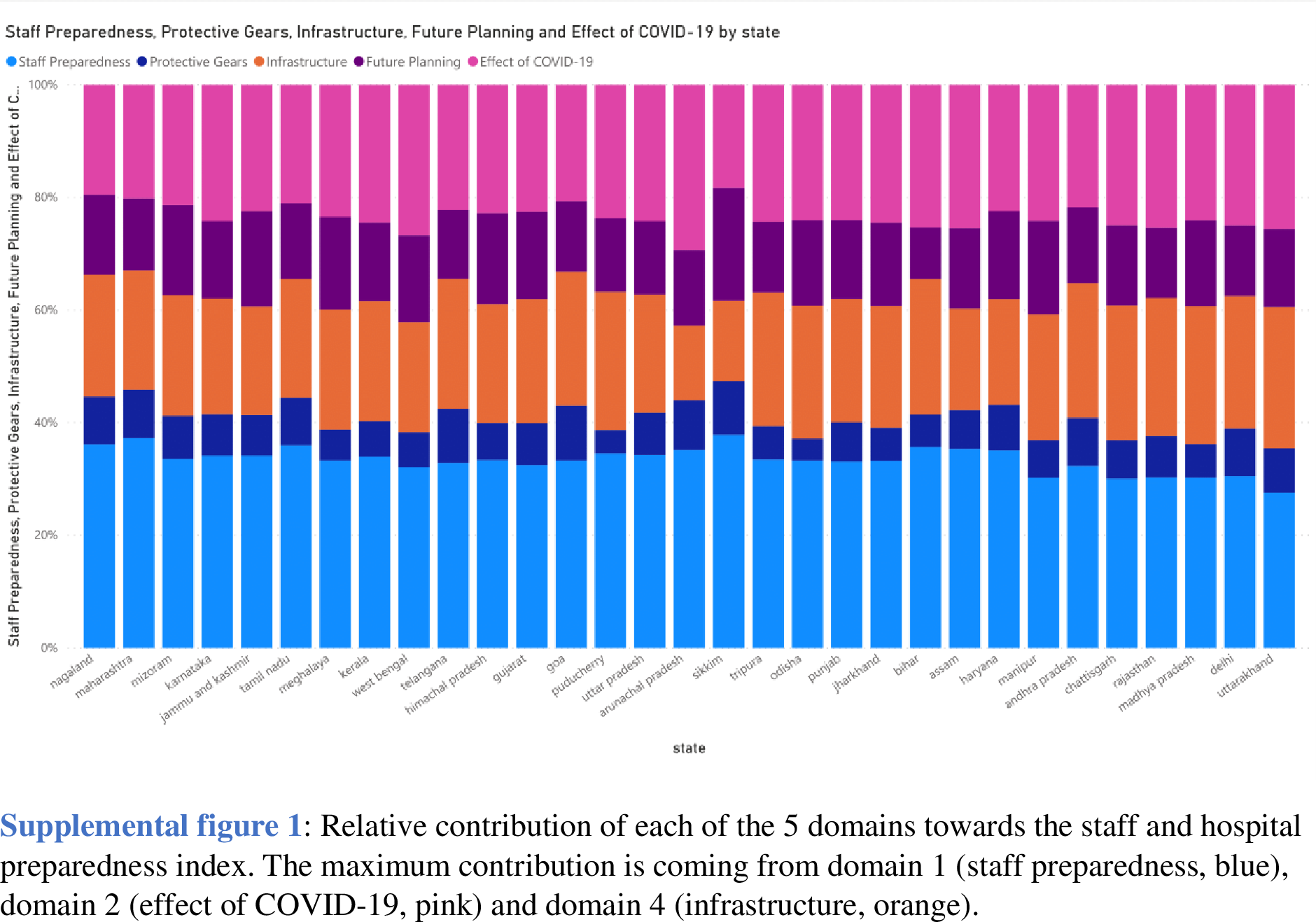

Supplement: S1 Fig — The maximum contribution is coming from domain 1 (staff preparedness, blue), domain 2 (effect of COVID-19, pink) and domain 4(infrastructure, orange). (TIF) [file pone.0269842.s004.tif]

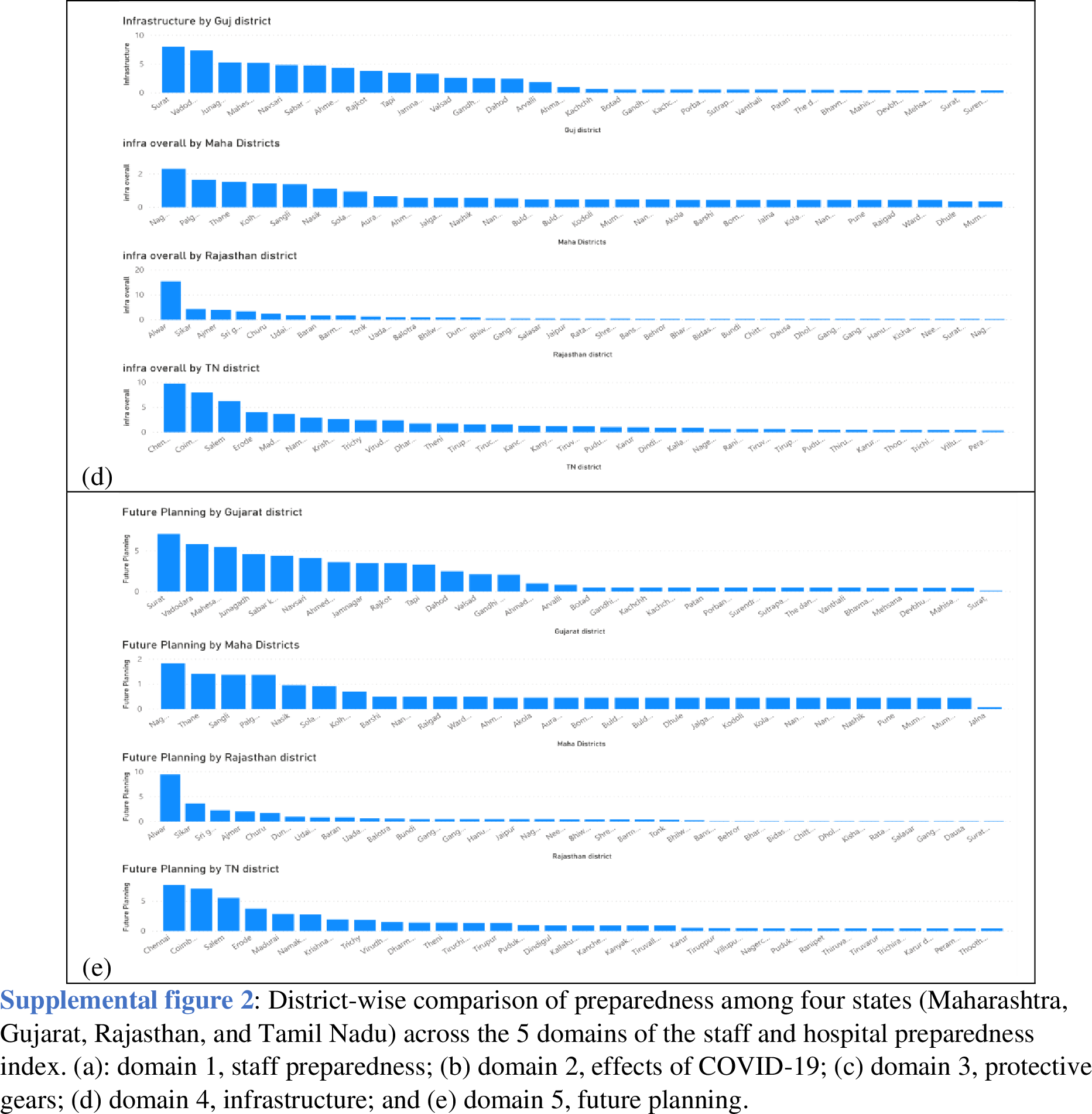

Supplement: S2 Fig — (a): domain 1, staff preparedness; (b) domain 2, effects of COVID-19; (c) domain 3, protective gears; (d) domain 4, infrastructure; and (e) domain 5, future planning. (TIF) [file pone.0269842.s005.tif]
