# Supplementary material for: Using a national level cross-sectional study to develop a Hospital Preparedness Index (HOSPI) for Covid-19 management: A case study from India
Source: PLoS One. 2022 Jul 27;17(7):e0269842. doi: 10.1371/journal.pone.0269842 (PMC9328545; doi:10.1371/journal.pone.0269842)
Supplement: S2 Table — (DOCX) [file pone.0269842.s003.docx]

**Supplemental table 2 (S2):** State-wise distribution of sample size

| **No** | **State/ UT** | **Total registered hospitals** | **Target**  **(5% response)** | **Achieved** | **% achieved** | **Remarks** |
| --- | --- | --- | --- | --- | --- | --- |
| 1 | Andhra Pradesh | 1779 | 89 | 16 | 18.0 | Included |
| 2 | Arunachal Pradesh | 20 | 1 | 1 | 100.0 | Clubbed with NE states |
| 3 | Assam | 390 | 20 | 21 | 105.0 | Included |
| 4 | Bihar | 871 | 44 | 43 | 97.7 | Included |
| 5 | Chhattisgarh | 334 | 17 | 19 | 111.8 | Included |
| 6 | Goa | 25 | 1 | 1 | 100.0 | Included |
| 7 | Gujarat | 2706 | 135 | 133 | 98.5 | Included |
| 8 | Haryana | 539 | 27 | 27 | 100.0 | Included |
| 9 | Himachal Pradesh | 215 | 11 | 11 | 100.0 | Included |
| 10 | Jammu and Kashmir | 230 | 12 | 12 | 100.0 | Included |
| 11 | Jharkhand | 757 | 38 | 47 | 123.7 | Included |
| 12 | Karnataka | 3423 | 171 | 168 | 98.2 | Included |
| 13 | Kerala | 405 | 20 | 20 | 100.0 | Included |
| 14 | Madhya Pradesh | 590 | 30 | 30 | 100.0 | Included |
| 15 | Maharashtra | 811 | 41 | 41 | 100.0 | Included |
| 16 | Manipur | 58 | 3 | 3 | 100.0 | Clubbed with NE states |
| 17 | Meghalaya | 179 | 9 | 9 | 100.0 | Clubbed with NE states |
| 18 | Mizoram | 101 | 5 | 5 | 100.0 | Clubbed with NE states |
| 19 | Nagaland | 101 | 5 | 5 | 100.0 | Clubbed with NE states |
| 20 | Odisha | 30 | 2 | 2 | 100.0 | Excluded: Not participating in ABY |
| 21 | Punjab | 771 | 39 | 39 | 100.0 | Included |
| 22 | Rajasthan | 2090 | 105 | 105 | 100.0 | Included |
| 23 | Sikkim | 12 | 1 | 1 | 100.0 | Clubbed with NE states |
| 24 | Tamil Nadu | 2334 | 117 | 117 | 100.0 | Included |
| 25 | Telangana | 21 | 1 | 1 | 100.0 | Excluded: Not participating in ABY |
| 26 | Tripura | 104 | 5 | 12 | 240.0 | Clubbed with NE states |
| 27 | Uttarakhand | 190 | 10 | 9 | 90.0 | Included |
| 28 | Uttar Pradesh | 1080 | 54 | 53 | 98.1 | Included |
| 29 | West Bengal | 61 | 3 | 4 | 133.3 | Excluded: Not participating in ABY |
| 30 | Chandigarh | 22 | 1 | 1 | 100.0 | Clubbed with Punjab |
| 31 | Delhi | 63 | 3 | 5 | 166.7 | Included |
| 32 | Puducherry | 24 | 1 | 1 | 100.0 | Included |
| 33 | Lakshadweep | 1 | 0 | 0 | 0.0 | Excluded: no response |
| 34 | Andaman and Nicobar Islands | 3 | 0 | 0 | 0.0 | Excluded: no response |
| 35 | Dadar and Nagar Haveli | 4 | 0 | 0 | 0.0 | Excluded: no response |
| 36 | Daman and Diu | 3 | 0 | 0 | 0.0 | Excluded: no response |
|  | **Total** | **20,347** | **1,021** | **962** | **94.2** |  |
|  | **Included in analysis** | **20,202** | **1,014** | **954** |  |  |
|  | **Excluded from analysis** | **145** | **7** | **8** |  |  |
